# SNW1 is a prognostic biomarker in prostate cancer

**DOI:** 10.1186/s13000-019-0810-8

**Published:** 2019-05-01

**Authors:** Doris Höflmayer, Carla Willich, Claudia Hube-Magg, Ronald Simon, Dagmar Lang, Emily Neubauer, Frank Jacobsen, Andrea Hinsch, Andreas M. Luebke, Marie Christina Tsourlakis, Hartwig Huland, Markus Graefen, Alexander Haese, Hans Heinzer, Sarah Minner, Franziska Büscheck, Guido Sauter, Thorsten Schlomm, Stefan Steurer, Till S. Clauditz, Eike Burandt, Waldemar Wilczak, Christian Bernreuther

**Affiliations:** 1 Institute of Pathology, University Medical Center Hamburg-Eppendorf, Martinistrasse 52, D-20246 Hamburg, Germany; 2 Martini-Clinic, Prostate Cancer Center, University Medical Center Hamburg-Eppendorf, Martinistrasse 52, D-20246 Hamburg, Germany; 3 Department of Urology, Charité - Universitätsmedizin Berlin, Charitéplatz 1, D-10117 Berlin, Germany

**Keywords:** SNW1, Prostate cancer, Prognosis, *TMPRSS2:ERG*, SKIP1

## Abstract

**Background:**

SNW1 is a nuclear receptor co-activator involved in splicing and transcription control, including androgen receptor signaling. Overexpression of SNW1 has been linked to adverse prognosis in different cancer types, but studies on the role of SNW1 in prostate cancer are lacking.

**Methods:**

Using immunohistochemistry, we analyzed SNW1 expression in 10,310 prostate cancers in a tissue microarray (TMA) with attached clinical and molecular data.

**Results:**

The comparison with normal prostate tissue revealed an up regulation of SNW1 in a subset of cancer samples. SNW1 staining was considered weak in 31.5%, moderate in 37.7% and strong in 14% of cancers. Strong SNW1 expression was markedly more frequent in prostate cancers harboring the *TMPRSS2:ERG* fusion (24%) than in ERG negative cancers (7%, *p* < 0.0001). Significant associations with Gleason grade, stage, nodal status and early biochemical recurrence were observed in the ERG negative and positive subset. Multivariable modeling revealed that the prognostic value of SNW1 up regulation was independent from the established preoperative histopathological and clinical parameters.

**Conclusion:**

These results demonstrate that SNW1 overexpression is an independent prognostic marker in prostate cancer with potential clinical utility.

**Electronic supplementary material:**

The online version of this article (10.1186/s13000-019-0810-8) contains supplementary material, which is available to authorized users.

## Background

Prostate cancer was estimated to be the most frequent diagnosed cancer and the third most common cause of cancer related death in men with western lifestyle in 2012 [1]. About 1 in 5 patients diagnosed with prostate cancer probably dies due to the cancer in developed countries. Established pretreatment prognostic parameters include Gleason grade and tumor extent on biopsies, preoperative prostate-specific antigen (PSA) levels, and clinical stage. Unfortunately, a reliable distinction between indolent and aggressive prostate cancer is still not possible in individual patients. It is hoped that upcoming molecular markers may enable a better prediction of prostate cancer aggressiveness.

SNW domain-containing protein 1 (SNW1) alias SKI-interacting protein 1 (SKIP1) is a multifunctional protein implicated in the regulation of several genes and pathways connected to cell growth and homeostasis. It acts either by direct protein interactions, by mRNA splicing regulation, or by transcriptional control. SNW1 has been originally named after its direct interaction with the SKI oncogene in the regulation TGF-ß signaling [2, 3], but it also associates with the retinoblastoma tumor suppressor protein to impair its function [4], and stabilizes beta-catenin to enhance WNT-signaling [5]. In its function as a selective splicing factor for p21, SNW1 is known to counteract p53-mediated apoptosis [6]. Finally, SNW1 is an important co-regulator for nuclear receptors [7] including vitamin D receptor [8], progesterone and androgen receptor [9]. Given these numerous interactions with pathways relevant in cancer, it is not surprising that SNW1 can be up regulated in many cancer types. For example, overexpression of SNW1 was reported in 43% of breast cancers [10], 50% of liver cancers [11], 30% of bladder cancers [12] and 40% of malignant pleural mesothelioma [13]. High SNW1 expression was associated with high tumor grade and proliferation in breast cancer [10] and with poor prognosis in the other above-mentioned tumor types [11–13]. Given its functional association with the androgen receptor, it appears possible that SNW1 expression may also be relevant in prostate cancer biology. Studies investigating the potential prognostic impact of SNW1 in clinical prostate cancer samples are currently missing.

To test the potential of SNW1 as a clinical marker in prostate cancer, the validated antibody HPA002457 from the Human Tissue Atlas Project [14] was employed for an immunohistochemical analysis of a highly annotated tissue micro array (TMA) with more than 12,000 prostate cancers.

## Methods

### Patients

The 12,427 patients had radical prostatectomy between 1992 and 2012 at the University Medical Center Hamburg-Eppendorf (Department of Urology and the Prostate Cancer Center Martini Clinic). Follow-up data were available for a total of 11,665 patients with a median follow-up of 60 months (range: 1 to 275 months; Table 1). PSA recurrence was defined as the time point at which postoperative serum PSA level was at least 0.2 ng/ml and increasing. Prostate specimens were analyzed according to a standard procedure [15]. In addition to the classical Gleason categories, “quantitative” Gleason grading was performed as detailed in Sauter et al. [16]. The TMA manufacturing process was described before [17]. The highly annotated database of this TMA contained results on ERG expression [18], ERG break-apart FISH analysis [19] and androgen receptor (AR) expression [20].

**Table 1:** Pathological and clinical data of the arrayed prostate cancers

	No. of patients (%)	
	Study cohort on TMA^a^	Biochemical relapse
Follow-up (month)		
N	11,665	2769 (23.7%)
Mean / median	62.9 / 50.0	–
Age (y)		
≤ 50	334	81 (24.3%)
51–59	3061	705 (23.0%)
60–69	7188	1610 (22.4%)
≥ 70	1761	370 (21.0%)
Pretreatment PSA (ng/ml)		
< 4	1585	242 (15.3%)
4–10	7480	1355 (18.1%)
10–20	2412	737 (30.6%)
> 20	812	397 (48.9%)
pT stage (AJCC 2002)		
pT2	8187	1095 (13.4%)
pT3a	2660	817 (30.7%)
pT3b-4	1528	847 (55.4%)
Gleason grade		
≤ 3 + 3	2848	234 (8.2%)
3 + 4	6679	1240 (18.6%)
3 + 4 Tertiary 5	433	115 (26.6%)
4 + 3	1210	576 (47.6%)
4 + 3 Tertiary 5	646	317 (49.1%)
≥ 4 + 4	596	348 (58.4%)
pN stage		
pN0	6970	1636 (23.5%)
pN+	693	393 (56.7%)
Surgical margin		
Negative	9990	1848 (18.5%)
Positive	2211	853 (38.6%)

^a^Numbers do not always add up to 12,427 in different categories because of cases with missing data. *Abbreviation: AJCC* American Joint Committee on Cancer

### Immunohistochemistry (IHC)

Freshly cut TMA sections were stained within one day in one assay. Slides were deparaffinized, rehydrated, washed in DAKO buffer (K8002) and transferred to a DAKO Link 48 autostainer. The polyclonal rabbit anti-SNW1 antibody HPA002457 supported by the Human Protein Atlas [14] was used at 1:150 dilution (HPA002457 Sigma, Merck, Darmstadt, Germany). The SNW1 staining was nuclear and paralleled by cytoplasmic co-staining of comparable intensity. The nuclear staining intensity (0, 1+, 2+, and 3+) and the fraction of positive tumor cells were separately recorded for each tissue spot. A final score was built of these two parameters according to the following score, which has been previously described [19]: Negative scores had a complete absence of nuclear and cytoplasmic staining; weak scores had a nuclear staining intensity of 1+ in ≤70% of the tumor cells or a staining intensity of 2+ in ≤30% of the tumor cells; moderate scores had a nuclear staining intensity of 1+ in > 70% of tumor cells, a staining intensity of 2+ in > 30% but in ≤70% of the tumor cells or a staining intensity of 3+ in ≤30% of the tumor cells; and strong scores had a nuclear staining intensity of 2+ in > 70% of the tumor cells or a staining intensity of 3+ in > 30% of the tumor cells. Scoring was done by two experienced pathologists with the help of a third one in cases with discordant results.

### Statistical analysis

Contingency tables and the χ^2^-test were performed to search for associations between molecular parameters and tumor phenotype. Analysis of variance was applied to search for differences of the KI67 labeling index (Ki67LI). Kaplan-Meier survival curves were compared by the log-rank test. Cox proportional hazards regression analysis was performed to test for independence and significance between pathological, molecular and clinical variables. Significant factors of the univariate models were covariates in the multivariate model. Calculations were performed with JMP 10 (SAS Institute Inc., NC, USA).

## Results

**Fig. 1:**
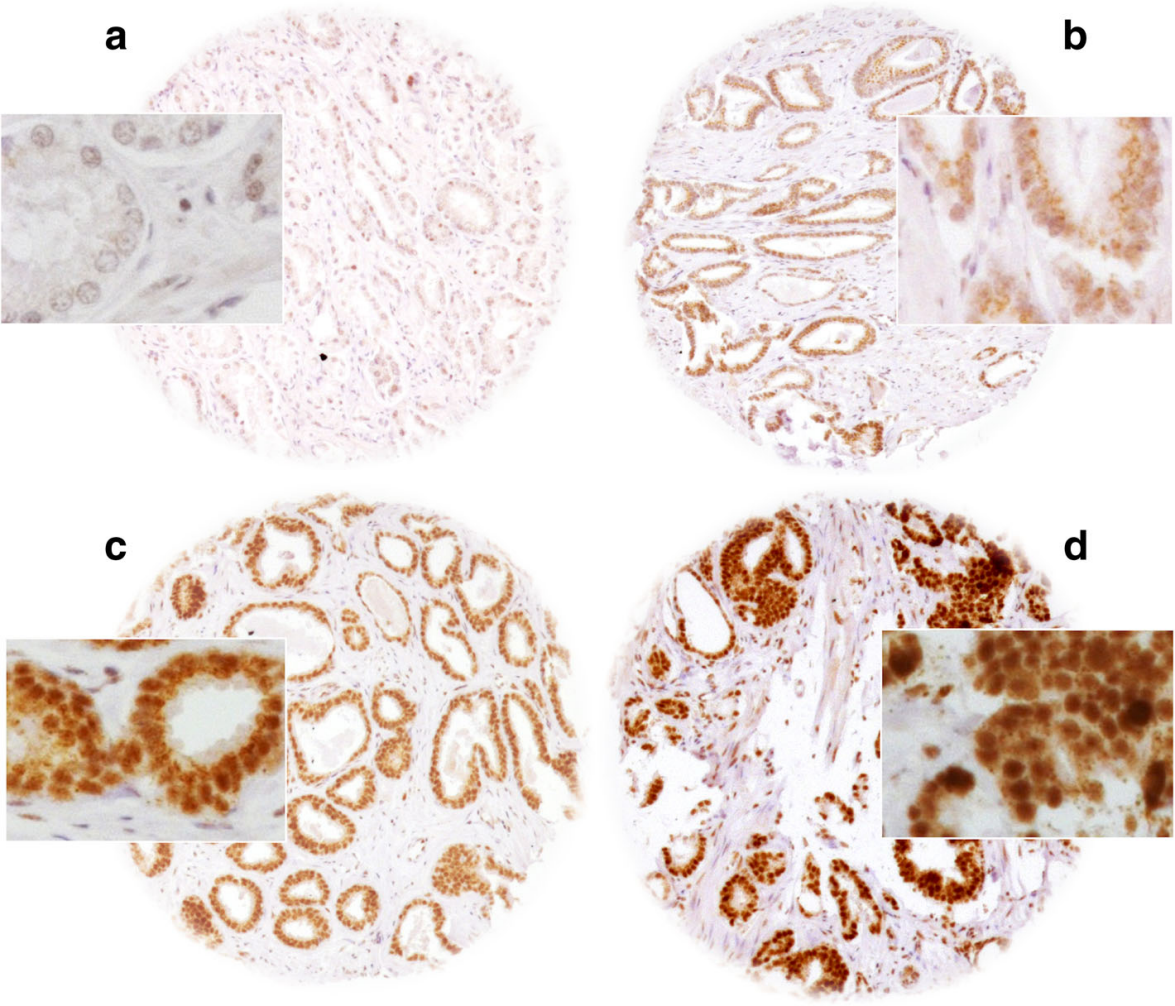
Representative pictures of **a**) negative, **b**) weak, **c**) moderate and **d**) strong SNW1 staining in prostate cancer. Spot size is 600 μm at 100x and 400x (inset) magnification

A total of 10,310 (83%) tumor samples were interpretable in our TMA analysis. Non-informative cases (2117; 17%) lacked tissue samples or unequivocal cancer cells in the TMA spot (Additional file 1: Table S1). Normal prostate glands showed variable levels of cytoplasmic and nuclear SNW1 staining, ranging between negative and moderate intensity (Additional file 1: Figure S1). In prostate cancers, positive SNW1 staining was found in 83.1% of cases, and was considered weak in 31.5%, moderate in 37.7% and strong in 14% of cases. Representative images are shown in Fig. 1.

### Association with *TMPRSS2:ERG* fusion status and ERG protein expression

**Fig. 2:**
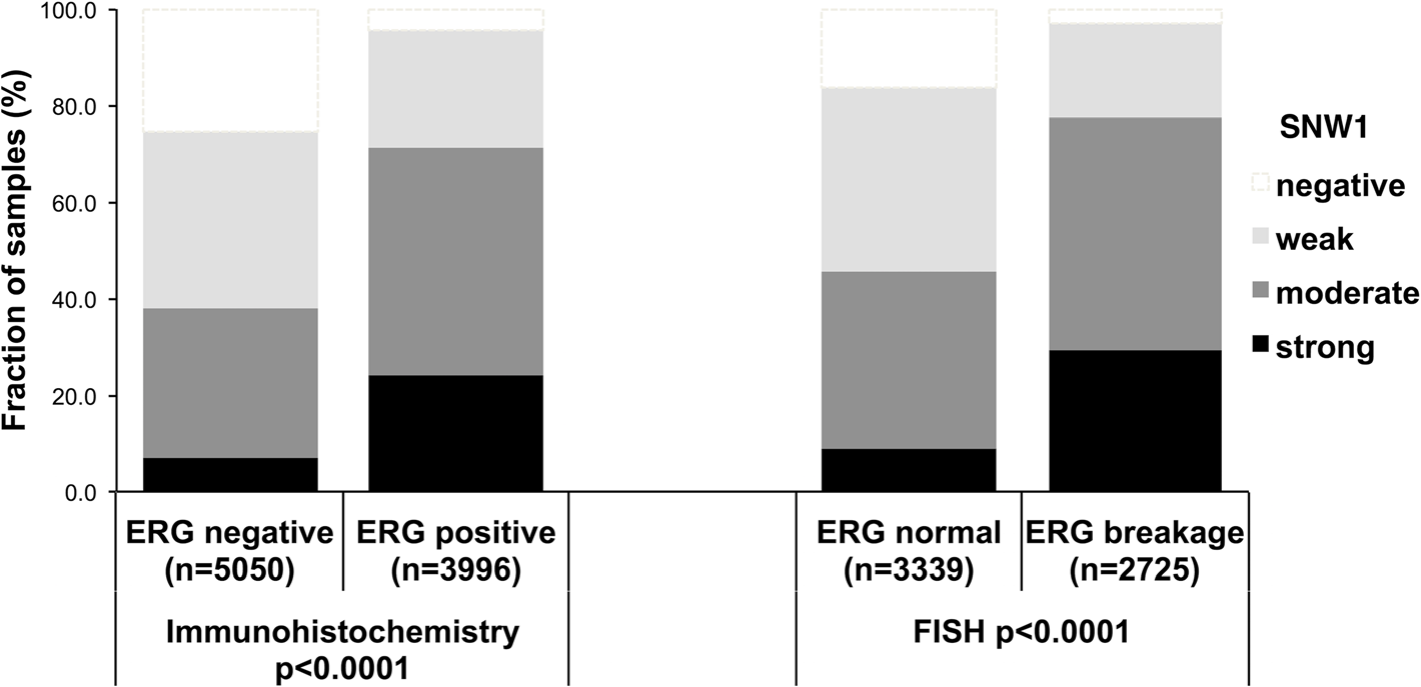
Association between positive SNW1 staining and ERG-status (Immunohistochemistry / FISH) in all cancers

To evaluate whether SNW1 expression is associated with ERG status in prostate cancers, we used data from previous studies (expanded from [18, 19]). Data on both ERG FISH and IHC were available from 6778 cancer samples and an identical result (ERG IHC positive and break by FISH or ERG IHC negative and missing break by FISH) was found in more than 95% of the examined cancer samples. SNW1 expression was strongly linked to *TMPRSS2:ERG* rearrangement and ERG expression in our set of prostate cancers. For example, moderate to strong SNW1 staining was seen in 37.9% of ERG-IHC negative but in 71.4% of ERG-IHC positive cancers (*p* < 0.0001). This was particularly evident for cancer samples with strong SNW1 expression, which was found in only 7% of ERG negative but in 24% of ERG positive cancers. All data are summarized in Fig. 2.

### Associations with tumor phenotype

Elevated SNW1 staining was linked to advanced tumor stage (< 0.0001), high classical or quantitative Gleason grade (< 0.0001 each), nodal metastasis (< 0.0001), and positive surgical margin (*p* = 0.0034) if all tumors were jointly analyzed (Table 2). Subset analysis of ERG fusion negative and positive cancers showed these associations in both subsets (Additional file 1: Table S2).

**Table 2:** Association between SNW1 staining and prostate cancer phenotype

		SNW1 (%)				
Parameter	N	Negative	Weak	Moderate	Strong	P
All cancers	10,310	16.9	31.5	37.7	14.0	
Tumor stage						< 0.0001
pT2	6668	18.7	33.2	36.3	11.8	
pT3a	2289	15.0	27.0	40.3	17.6	
pT3b-pT4	1313	10.2	30.2	40.1	19.5	
Gleason grade						< 0.0001
≤ 3 + 3	2302	25.0	34.2	31.9	8.9	
3 + 4	5275	16.1	31.2	38.0	14.7	
3 + 4 Tertiary 5	382	13.6	31.2	43.5	11.8	
4 + 3	916	12.2	27.9	41.2	18.7	
4 + 3 Tertiary 5	564	9.8	25.2	41.8	23.2	
≥ 4 + 4	420	7.9	28.1	46.2	17.9	
Lymph node metastasis						< 0.0001
N0	5818	16.0	30.4	38.5	15.1	
N+	591	8.3	27.6	45.2	19.0	
Preoperative PSA level (ng/ml)						0.0011
< 4	1278	13.2	30.0	41.6	15.2	
4–10	6160	16.8	31.4	37.4	14.4	
10–20	2047	18.1	32.6	36.3	13.0	
> 20	713	19.1	31.0	37.4	12.5	
Surgical margin						0.0034
Negative	8178	17.4	31.7	37.5	13.5	
Positive	1947	14.8	30.8	38.4	16.0	

### Association with tumor cell proliferation

High-level SNW1 staining was significantly linked to high cell proliferation as measured by Ki67LI. The average Ki67LI increased from 1.33 ± 0.08 in cancers lacking SNW1 expression to 2.55 ± 0.06 (weak), 3.25 ± 0.05 (moderate) and to 3.8 ± 0.09 in cancers with strong SNW1 expression (*p* < 0.0001). This association held true in all tumor subsets with identical Gleason score (≤3 + 3: *p* < 0.001, 3 + 4: *p* < 0.0001, 4 + 3: *p* < 0.0001, ≥4 + 4: *p* = 0.0060; data not shown). This relationship was more prominent in ERG negative than in ERG positive cancers (Additional file 1: Tables S3 and S4).

### Association with androgen receptor

**Fig. 3:**
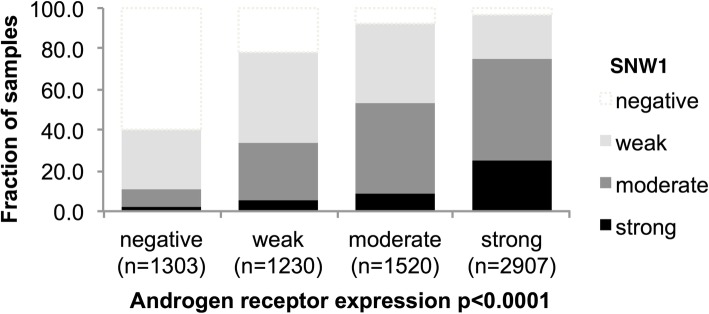
Association between SNW1 and androgen receptor expression

Because SNW1 is a known co-factor of androgen receptor signaling [9], we compared SNW1 expression with immunohistochemistry data on AR expression from a previous study [20]. Immunohistochemistry data on both SNW1 and AR were available from 6984 cancers. There was a strong and linear increase of SNW1 with increasing AR expression: Only 2% of AR-negative, but 25% of cancers with strong AR expression showed strong SNW1 expression (*p* < 0.0001; Fig. 3).

### Association with PSA recurrence

**Fig. 4:**
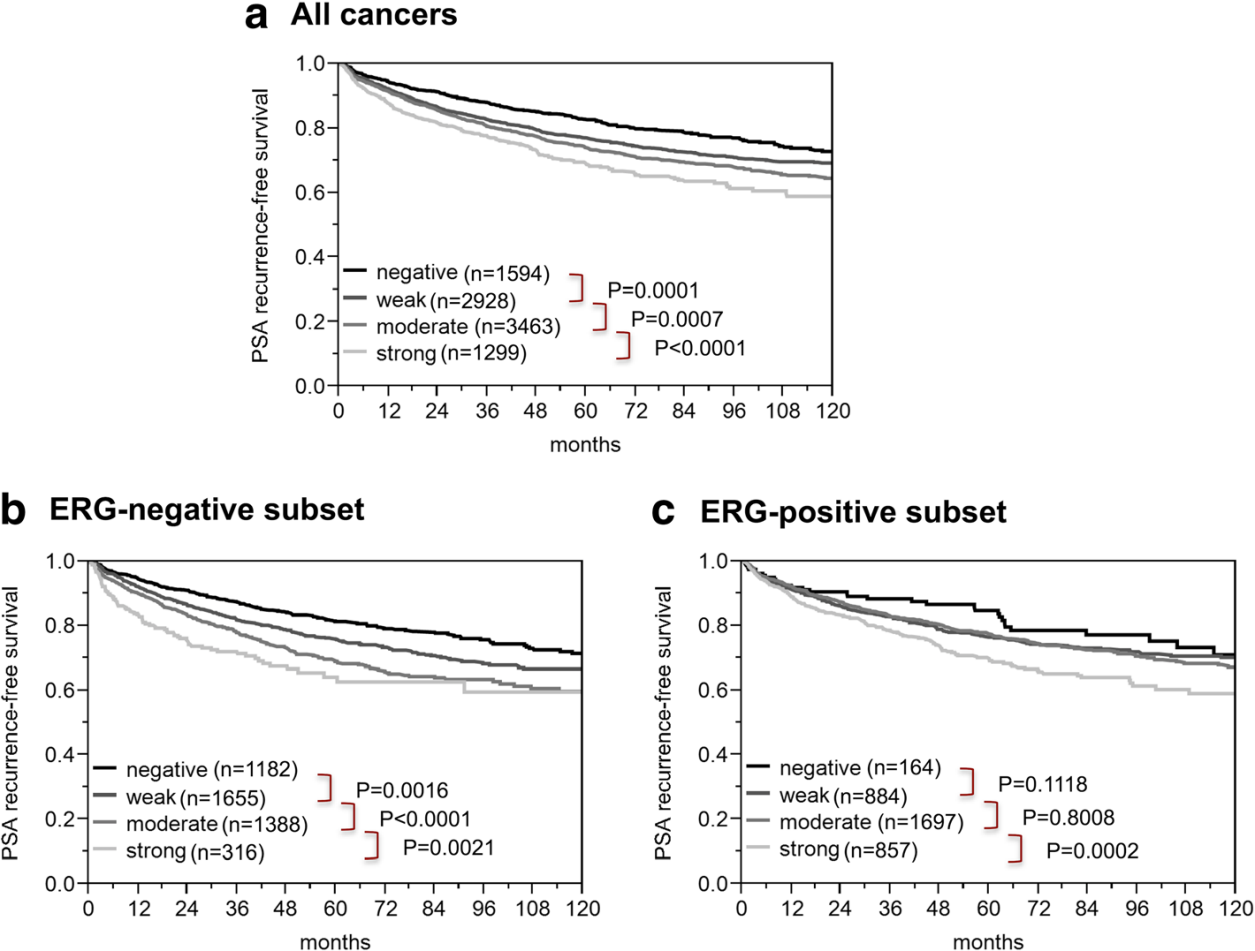
Association between SNW1 expression and biochemical recurrence in **a**) all cancers, **b**) the ERG-fusion negative, and **c**) ERG-fusion positive subsets

Follow-up data were available for 9284 patients with interpretable SNW1 staining on the TMA. High-level SNW1 expression was associated with early PSA recurrence (*p* < 0.0001, Fig. 4a). Subgroup analyses of ERG negative (*p* < 0.0001, Fig. 4b) and ERG positive cancers (*p* = 0.0002, Fig. 4c) revealed that the prognostic impact of SNW1 was significant in both subsets. To estimate the impact of the Gleason grade on the prognostic power of SNW1, we performed further subset analyses in ERG negative cancers with identical classical and quantitative Gleason grade. Here, SNW1 staining did not provide significant prognostic information beyond the classical or quantitative Gleason grade (Additional file 1: Figure S2).

### Uni- and multivariable analysis

Table 3 shows the hazard ratios for the established preoperative risk factors Gleason grade obtained on the original biopsy, preoperative PSA level, and cT stage together with the SNW1 expression ranked by their decreasing Cox proportional hazard in the uni- and multivariable model. SNW1 expression had a moderate effect in both models. The effect was seen in the ERG negative and ERG positive subset, although it was weaker in the later.

**Table 3:** Cox proportional hazards for PSA recurrence-free survival after prostatectomy of established preoperative prognostic parameter and SNW1 expression

Variable	Univariable analysis	Multivariable analysis
Gleason grade biopsy		
≥ 4 + 4 vs. ≤3 + 3	6.01 (5.41–6.66) ***	4.05 (3.58–4.58) ***
Preoperative PSA-level (ng/μl)		
> 20 vs. < 4	5.12 (4.46–5.89) ***	3.61 (3.00–4.35) ***
cT-stage		
T2b vs. T1c	2.16 (1.73–2.66) ***	1.84 (1.62–2.08) ***
SNW1 expression		
Strong vs. negative	1.95 (1.69–1.26) ***	1.63 (1.40–1.89) ***
ERG negative subset	2.38 (1.90–2.97) ***	1.88 (1.49–2.35) ***
ERG positive subset	1.81 (1.30–2.60) **	1.48 (1.06–2.14) *

The multivariable model included all the four-univariate factors. Confidence interval (95%) in brackets; asterisk indicate significance level: * *p* ≤ 0.05, ** *p* ≤ 0.001, *** *p* ≤ 0.0001; ERG ETS-related gene

## Discussion

The results of our study demonstrate that SNW1 overexpression is linked to aggressive tumor features and poor prognosis in prostate cancers. Our immunohistochemical analysis revealed at least weak SNW1 staining in 83% of our 10,310 interpretable prostate cancers. Normal prostate glands showed variable levels of SNW1 expression, ranging from negative to moderate staining in our study. That a strong SNW1 immunostaining - at a level not observed in normal prostate epithelium - was found in 14% of prostate cancers suggests that SNW1 becomes up regulated during tumor development in a fraction of prostate cancers. Comparable data derived from immunohistochemical analyses of SNW1 in prostate cancer are currently lacking in the literature. Data from RNA sequencing of prostate cancers do not show major SNW1 mRNA expression changes [21], suggesting that stabilization may account for the increased levels identified by immunohistochemistry. However, data supporting SNW1 up regulation in malignant tumors comes from breast cancer [10], hepatocellular carcinoma [11], bladder cancer [12] and malignant pleural mesothelioma [13].

Highly significant statistical associations of SNW1 overexpression with unfavorable tumor phenotype, accelerated cell proliferation and poor clinical outcome in our cohort of more than 10,000 patients identifies SNW overexpression as a sign for increased cancer aggressiveness. These observations fit well to the role of SNW1 as co-activator of known oncogenic pathways [2, 3], such as transforming growth factor β (TGF-β) [2] and WNT signaling [5]. Functional studies have further demonstrated, that SNW1 counteracts p53-mediated apoptosis [6] and inhibits the transcriptional repressor activity of the retinoblastoma tumor suppressor gene [4]. Accordingly, associations of SNW1 up regulation with unfavorable tumor features have also been reported from several other malignancies. For example, SNW1 overexpression is linked to high tumor grade and accelerated cell proliferation in breast cancer [10], and to poor prognosis in urinary bladder cancer, hepatocellular carcinoma, and malignant pleural mesothelioma [11–13].

The availability of a molecular database attached to this prostate cancer TMA enabled us to investigate the role of SNW1 in molecularly defined cancer subgroups, the most relevant of which are ERG positive and ERG negative cancers. *TMPRSS2:ERG* fusions occur in about 50% of prostate cancers [22]. They predominantly occur in younger patients [18] and lead to a constitutive overexpression of the transcription factor ERG [22]. ERG overexpression by itself lacks any prognostic impact, at least in patients not receiving systemic therapy. However, ERG modulates the expression of more than 1600 genes in prostate epithelial cells ERG [23–25]. The biological effects of various proteins may be mitigated or intensified in such a modified cellular microenvironment. Our data identify SNW1 expression as an ERG-dependent feature being clearly more prominent in ERG positive than in ERG negative cancers. It appears possible, that SNW1 up regulation in ERG-fusion positive tumors is mediated by ERG-dependent activation of TGF-ß signaling. Earlier studies have shown that TGF-ß signaling is massively up regulated in ERG-positive cancers [24], and that TGF-ß1 increases SNW1 expression in mouse models [26]. The strong association between SNW1 overexpression and increased AR expression observed in our study fits well to earlier reports on a functional relationship between these proteins. For example, Abwanka et al. [9] demonstrated that binding of testosterone to the AR and nuclear translocation of activated AR is massively increased when SNW1 forms complexes with the AR.

The prognostic effect of SNW1 expression was stronger in ERG negative than in ERG positive cancers but also retained in the latter group. A modified cellular microenvironment may serve as an explanation for the particularly strong prognostic role of SNW1 expression in ERG negative cancers. In earlier studies we had described several molecular features that were either prognostic in ERG positive (for example, SOX9, [27] and SENP1 [28] or in ERG negative cancers (for example, GGH [29] and NBS1 [30]) but not in both groups. An alternative explanation for different prognostic effects between ERG positive and ERG negative cancers is the experimental set-up. It cannot be excluded that our immunohistochemistry protocol was better suited to distinguish expression differences in cancers with generally lower expression levels (ERG negative cancers) than in those with higher expression (ERG positive cancers). For example, the group of SNW1 negative cancers – the best prognostic group – contained 1182 cancers in ERG negative, but only 164 cancers in ERG positive cancers. Irrespective of its cause, the different prognostic impact of SNW1 in ERG positive and ERG negative cancers demonstrates that the applicability (and perhaps thresholds) of prognostic markers may depend on individual tumor features. This clearly represents a challenge for the development of prognostic cancer tests that shall be applicable to every patient.

The data of this study suggest that SNW1 expression may represent a clinically useful marker. It is of note that the search for clinically useful prognostic markers is not predominantly about finding factors that are independent of established parameters. Most of all, parameters are needed that are more reproducible and, thus, reliable in individual patients. The established prognostic factors (Gleason grade, preoperative PSA-level, clinical stage, pathological stage and nodal status) are statistically strong but suffer from significant shortcomings in clinical practice. pT stage and nodal status are not available during the preoperative therapeutic decision-making process. The quality of pN data greatly depends on the extent of surgery and the pathological work-up of the removed materials. The Gleason Grade – i.e., the most powerful preoperatively available prognostic marker - suffers from very substantial inter-observer variability reaching beyond 40% in individual biopsies [31]. SNW1 expression measurement thus clearly has potential to become an element in a future multi-parametric prognostic test for prostate cancer. To this end some of the limitations in the present study (retrospective analysis of a single spot per patient from prostatectomy specimen) have to be addressed.

## Conclusions

SNW1 overexpression occurred in a relevant fraction of prostate cancers. The moderate prognostic impact of SNW1 makes it a candidate for future marker panels in prostate cancer.

## Additional file

Additional file 1: **Table S1.** Distribution of clinical parameters of patients with evaluable and not evaluable SNW1 staining results. **Table S2.** Association between SNW1 staining results and prostate cancer phenotype in ERG negative and ERG positive cancers. **Table S3.** Association between SNW1 staining in ERG negative cancers and tumor cell proliferation measured by Ki67 labeling index. **Table S4.** Association between SNW1 staining in ERG positive cancers and tumor cell proliferation measured by Ki67 labeling index. **Figure S1.** SNW1 staining in a heterogeneous TMA spot with benign glands (*) and carcinogenic glands (x) at 100/400x magnification. **Figure S2.** Prognostic impact of negative or strong SNW1 expression in subsets of cancers defined by a) the classical Gleason score (black dotted lines) and b-h) the quantitative Gleason score categories (black dotted lines) defined by the percentage of Gleason 4 patterns: b) ≤5%, c) 6–10%, d) 11–20%, e) 21–30%, f) 31–49%, g) 50–60%, and h) 61–100% Gleason 4 pattern. (DOCX 9807 kb)

## Abbreviations

AR: Androgen receptor
ERG: Erythroblast transformation-specific (ETS) related gene
FISH: Fluorescence in situ hybridization
IHC: Immunohistochemistry
Ki67LI: Ki67 labeling index
PSA: Prostate specific antigen
SNW1: SNW domain-containing protein 1
TGF-β: Transforming growth factor β
TMA: Tissue microarray
TMPRSS2: Trans membrane protease, serine 2
WNT: Wingless Int-1
