# Supplementary material for: SNW1 is a prognostic biomarker in prostate cancer
Source: Diagn Pathol. 2019 May 1;14:33. doi: 10.1186/s13000-019-0810-8 (PMC6495565; doi:10.1186/s13000-019-0810-8)
Supplement: Supplementary file 1 — Table S1. Distribution of clinical parameters of patients with evaluable and not evaluable SNW1 staining results. Table S2. Association between SNW1 staining results and prostate cancer phenotype in ERG negative and ERG positive cancers. Table S3. Association between SNW1 staining in ERG negative cancers and tumor cell proliferation measured by Ki67 labeling index. Table S4. Association between SNW1 staining in ERG positive cancers and tumor cell proliferation measured by Ki67 labeling index. Figure S1. SNW1 staining in a heterogeneous TMA spot with benign glands (*) and carcinogenic glands (x) at 100/400x magnification. Figure S2. Prognostic impact of negative or strong SNW1 expression in subsets of cancers defined by a) the classical Gleason score (black dotted lines) and b-h) the quantitative Gleason score categories (black dotted lines) defined by the percentage of Gleason 4 patterns: b) ≤5%, c) 6–10%, d) 11–20%, e) 21–30%, f) 31–49%, g) 50–60%, and h) 61–100% Gleason 4 pattern. (DOCX 9807 kb) [file 13000_2019_810_MOESM1_ESM.docx]

**Supplementary Information**

**Table S1** Distribution of clinical parameters of patients with evaluable and not evaluable SNW1 staining

| **Parameter** | **Evaluable** | **Not evaluable** |
| --- | --- | --- |
| Tumor stage | N=10,310* | N=2,117* |
| pT2 | 64.9 | 72.1 |
| pT3a | 22.3 | 17.7 |
| pT3b-4 | 12.8 | 10.2 |
|  |  |  |
| Gleason grade | N=10,303 | N=2,110 |
| ≤3+3 | 22.4 | 26.3 |
| 3+4 | 53.8 | 53.3 |
| 3+4 Tert.5 | 3.7 | 2.5 |
| 4+3 | 10.0 | 8.2 |
| 4+3 Tert.5 | 5.5 | 4.1 |
| ≥4+4 | 4.6 | 5.7 |
|  |  |  |
| Lymph node metastasis | N=10,250 | N=2,107 |
| N0 | 56.8 | 54.8 |
| N+ | 43.3 | 45.2 |
|  |  |  |
| Preoperative PSA | N=10,235 | N=2,103 |
| <4 | 12.6 | 14.7 |
| 4-10 | 60.4 | 63.0 |
| 11-20 | 20.1 | 17.5 |
| >20 | 7.0 | 4.8 |
|  |  |  |
| Surgical margin | N=10,268 | N=2,109 |
| Negative | 79.8 | 86.1 |
| Positive | 20.3 | 13.9 |

* With missing values total numbers in categories add up to 12,427

**Table S2** Association between SNW1 staining results and prostate cancer phenotype in ERG negative and ERG positive cancers

|  |  | **SNW1 (%) in ERG-negative cancers** | | | |  | |  | |  | **SNW1 (%) in ERG-positive cancers** | | | | | | | | |  | |  |
| --- | --- | --- | --- | --- | --- | --- | --- | --- | --- | --- | --- | --- | --- | --- | --- | --- | --- | --- | --- | --- | --- | --- |
| **Parameter** | **N** | **Negative** | **Weak** | **Moderate** | **Strong** | **P** |  | | **N** | | **Negative** | | **Weak** | | **Moderate** | | **Strong** | | **P** | |  |  |
| **All cancers** | 5050 | 25.5 | 36.7 | 30.9 | 7.0 |  |  | | 3996 | | 4.4 | | 24.1 | | 47.4 | | 24.0 | |  | |  |  |
| **Tumor stage** |  |  |  |  |  | <0.0001 |  | |  | |  | |  | |  | |  | | <0.0001 | |  |  |
| pT2 | 3389 | 27.5 | 38.0 | 29.0 | 5.5 |  |  | | 2372 | | 4.6 | | 25.7 | | 48.1 | | 21.7 | |  |  |  |  |
| pT3a | 1005 | 24.9 | 34.0 | 32.8 | 8.3 |  |  | | 1067 | | 4.5 | | 20.0 | | 48.8 | | 26.7 | |  |  |  |  |
| pT3b-pT4 | 642 | 15.4 | 33.6 | 37.7 | 13.2 |  |  | | 540 | | 3.3 | | 25.4 | | 41.9 | | 29.4 | |  |  |  |  |
| **Quantitative Gleason grade** | | | | | | | |  | |  | |  | |  | |  | |  | |  | | |
| ≤3+3 | 1036 | 37.6 | 36.0 | 22.2 | 4.2 | p<0.0001 |  | | 838 | | 7.8 | | 30.5 | | 45.7 | | 16.0 | | p<0.0001 | |  |  |
| 3+4 ≤5% | 724 | 27.1 | 38.0 | 29.3 | 5.7 |  |  | | 572 | | 5.1 | | 28.1 | | 46.3 | | 20.5 | |  |  |  |  |
| 3+4 6-10% | 689 | 27.0 | 39.2 | 29.2 | 4.6 |  |  | | 603 | | 2.8 | | 19.4 | | 51.7 | | 26.0 | |  |  |  |  |
| 3+4 11-20% | 592 | 23.8 | 36.3 | 33.3 | 6.6 |  |  | | 480 | | 3.5 | | 22.1 | | 48.3 | | 26.0 | |  |  |  |  |
| 3+4 21-30% | 304 | 25.0 | 35.5 | 30.6 | 8.9 |  |  | | 289 | | 3.8 | | 24.9 | | 44.6 | | 26.6 | |  |  |  |  |
| 3+4 31-49% | 261 | 22.6 | 38.3 | 29.5 | 9.6 |  |  | | 210 | | 4.8 | | 20.0 | | 45.7 | | 29.5 | |  |  |  |  |
| 3+4 Tertiary 5 | 232 | 19.0 | 39.7 | 35.8 | 5.6 |  |  | | 122 | | 1.6 | | 18.0 | | 56.6 | | 23.8 | |  |  |  |  |
| 4+3 50-60% | 218 | 20.2 | 35.8 | 34.4 | 9.6 |  |  | | 173 | | 3.5 | | 16.8 | | 48.0 | | 31.8 | |  |  |  |  |
| 4+3 61-80% | 207 | 17.9 | 31.9 | 40.1 | 10.1 |  |  | | 145 | | 3.4 | | 20.0 | | 48.3 | | 28.3 | |  |  |  |  |
| 4+3 >80% | 57 | 14.0 | 40.4 | 29.8 | 15.8 |  |  | | 31 | | 3.2 | | 22.6 | | 41.9 | | 32.3 | |  |  |  |  |
| 4+3 Tertiary 5 | 300 | 15.7 | 30.3 | 39.7 | 14.3 |  |  | | 224 | | 1.8 | | 16.1 | | 45.1 | | 37.1 | |  |  |  |  |
| ≥4+4 | 253 | 10.6 | 31.5 | 46.1 | 11.8 |  |  | | 124 | | 1.6 | | 18.5 | | 45.2 | | 34.7 | |  |  |  |  |
| **Lymph node metastasis** | | | |  |  | <0.0001 |  | |  | |  | |  | |  | |  | | 0.4323 | |  |  |
| N0 | 2948 | 24.1 | 36.3 | 32.2 | 7.5 |  |  | | 2280 | | 4.1 | | 22.5 | | 47.5 | | 25.9 | |  |  |  |  |
| N+ | 283 | 13.1 | 28.6 | 43.8 | 14.5 |  |  | | 249 | | 2.4 | | 25.3 | | 45.8 | | 26.5 | |  |  |  |  |
| **Preoperative PSA level (ng/ml)** | | | | | | 0.0143 |  | |  | |  | |  | |  | |  | | 0.7997 | |  |  |
| <4 | 530 | 20.2 | 34.7 | 36.0 | 9.1 |  |  | | 562 | | 4.4 | | 24.9 | | 47.3 | | 23.3 | |  |  |  |  |
| 4-10 | 2969 | 25.3 | 37.4 | 30.6 | 6.6 |  |  | | 2423 | | 4.5 | | 23.6 | | 47.0 | | 24.8 | |  |  |  |  |
| 10-20 | 1103 | 26.8 | 37.0 | 28.8 | 7.3 |  |  | | 727 | | 3.9 | | 23.9 | | 50.1 | | 22.1 | |  |  |  |  |
| >20 | 405 | 28.1 | 32.8 | 32.1 | 6.9 |  |  | | 237 | | 4.6 | | 27.0 | | 43.9 | | 24.5 | |  |  |  |  |
| **Surgical margin** | | | | |  | 0.0254 |  | |  | |  | |  | |  | |  | | 0.2861 | |  |  |
| Negative | 4008 | 26.1 | 37.0 | 30.1 | 6.7 |  |  | | 3131 | | 4.5 | | 24.4 | | 47.8 | | 23.2 | |  |  |  |  |
| Positive | 958 | 22.8 | 35.4 | 33.8 | 8.0 |  |  | | 793 | | 3.8 | | 24.2 | | 45.8 | | 26.2 | |  |  |  |  |

**Table S3** Association between SNW1 staining in ERG *negative* cancers and tumor cell proliferation measured by Ki67 labeling index

| **Gleason** | **SNW1** | **N** | **Ki67LI (mean±SEM)** | **P** |
| --- | --- | --- | --- | --- |
| **Total** | negative | 953 | 1.29±0.08 | <0.0001 |
|  | weak | 1191 | 2.69±0.08 |  |
|  | moderate | 1001 | 3.56±0.08 |  |
|  | strong | 197 | 4.53±0.19 |  |
|  |  |  |  |  |
| **≤3+3** | negative | 289 | 1.20±0.12 | <0.0001 |
|  | weak | 230 | 2.37±0.14 |  |
|  | moderate | 129 | 2.95±0.18 |  |
|  | strong | 25 | 3.72±0.42 |  |
|  |  |  |  |  |
| **3+4** | negative | 491 | 1.18±0.10 | <0.0001 |
|  | weak | 664 | 2.50+0.08 |  |
|  | moderate | 525 | 3.12+0.09 |  |
|  | strong | 91 | 3.57+0.23 |  |
|  |  |  |  |  |
| **3+4 Tert.5** | negative | 33 | 1.70±1.70 | 0.0031 |
|  | weak | 60 | 3.33±3.33 |  |
|  | moderate | 62 | 3.69±3.69 |  |
|  | strong | 7 | 3.14±3.14 |  |
|  |  |  |  |  |
| **4+3** | negative | 81 | 1.68+0.38 | <0.0001 |
|  | weak | 121 | 3.36+0.31 |  |
|  | moderate | 129 | 3.98+0.30 |  |
|  | strong | 33 | 6.18+0.59 |  |
|  |  |  |  |  |
| **4+3 Tert.5** | negative | 34 | 1.68+0.65 | <0.0001 |
|  | weak | 58 | 3.24+0.50 |  |
|  | moderate | 79 | 4.81+0.42 |  |
|  | strong | 23 | 6.22+0.79 |  |
|  |  |  |  |  |
| **≥4+4** | negative | 25 | 2.00+0.79 | 0.0002 |
|  | weak | 58 | 3.50+0.52 |  |
|  | moderate | 77 | 5.47+0.45 |  |
|  | strong | 18 | 5.89+0.93 |  |

SEM, standard error of the mean

**Table S4** Association between SNW1 staining in ERG *positive* cancers and tumor cell proliferation measured by Ki67 labeling index

| **Gleason** | **SNW1** | **N** | **Ki67LI (mean±SEM)** | **P** |
| --- | --- | --- | --- | --- |
| **Total** | negative | 125 | 1.68±0.22 | <0.0001 |
|  | weak | 667 | 2.32±0.10 |  |
|  | moderate | 1363 | 3.02±0.07 |  |
|  | strong | 627 | 3.54±0.10 |  |
|  |  |  |  |  |
| **≤3+3** | negative | 49 | 1.57±0.27 | <0.0001 |
|  | weak | 188 | 2.11±0.14 |  |
|  | moderate | 290 | 2.58±0.11 |  |
|  | strong | 91 | 3.01±0.20 |  |
|  |  |  |  |  |
| **3+4** | negative | 62 | 1.55+0.29 | <0.0001 |
|  | weak | 372 | 2.19+0.12 |  |
|  | moderate | 802 | 3.07+0.08 |  |
|  | strong | 400 | 3.42+0.12 |  |
|  |  |  |  |  |
| **3+4 Tert.5** | negative | 1 | 10.0±2.43 | 0.0032 |
|  | weak | 17 | 2.24±0.59 |  |
|  | moderate | 49 | 3.43±0.35 |  |
|  | strong | 21 | 4.43±0.53 |  |
|  |  |  |  |  |
| **4+3** | negative | 9 | 1.33+0.88 | 0.0767 |
|  | weak | 53 | 2.87+0.36 |  |
|  | moderate | 127 | 3.31+0.24 |  |
|  | strong | 51 | 3.65+0.37 |  |
|  |  |  |  |  |
| **4+3 Tert.5** | negative | 2 | 2.00±2.46 | 0.0816 |
|  | weak | 21 | 4.10±0.76 |  |
|  | moderate | 60 | 2.82±0.45 |  |
|  | strong | 43 | 4.51±0.53 |  |
|  |  |  |  |  |
| **≥4+4** | negative | 2 | 5.50±3.65 | 0.0816 |
|  | weak | 16 | 3.81±1.29 |  |
|  | moderate | 35 | 4.23±0.87 |  |
|  | strong | 21 | 5.00±1.13 |  |

SEM, standard error of the mean


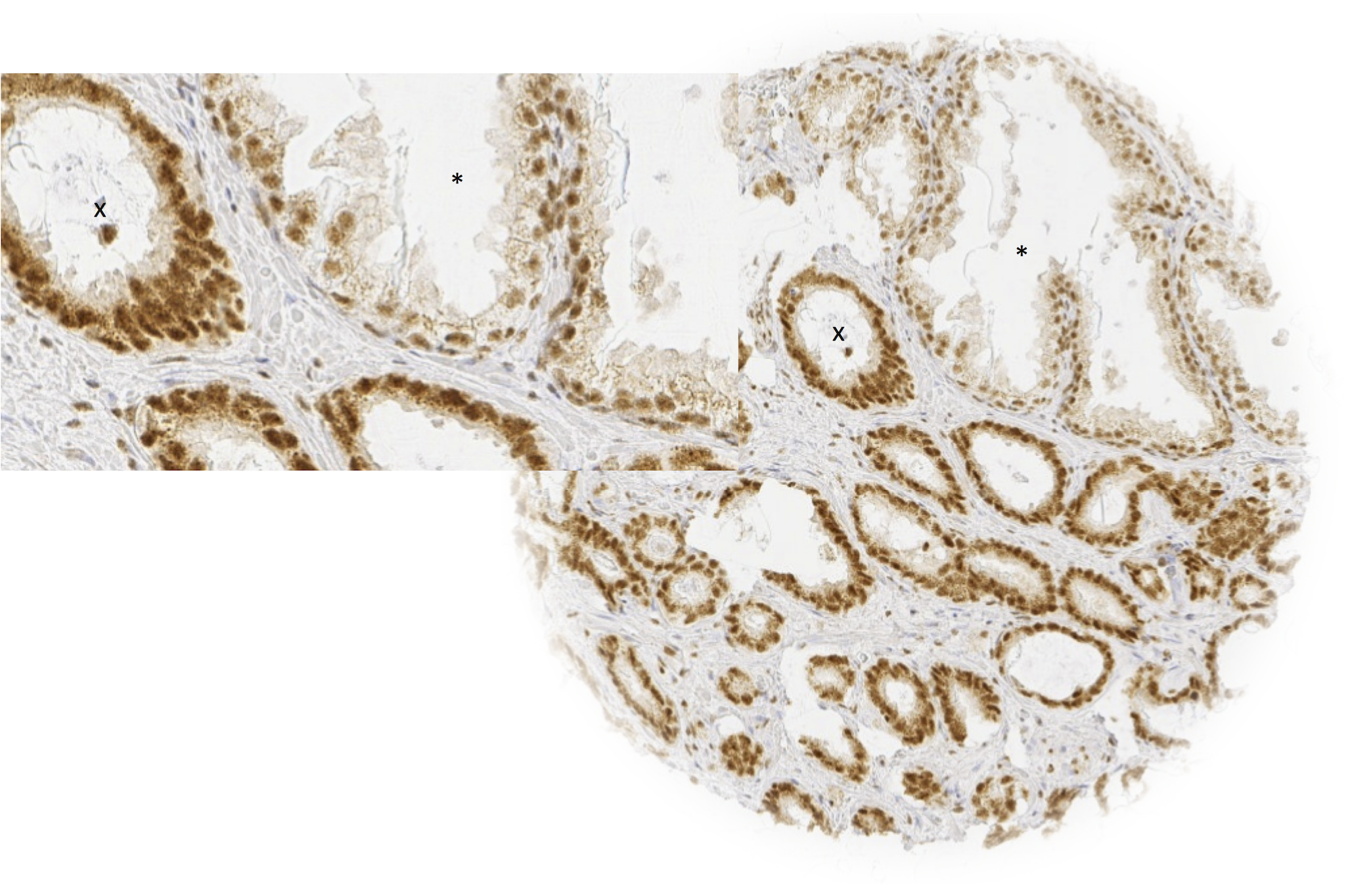


**Fig. S1** SNW1 staining in a heterogeneous TMA spot with benign glands (*) and carcinogenic glands (x) at 100/400x magnification.


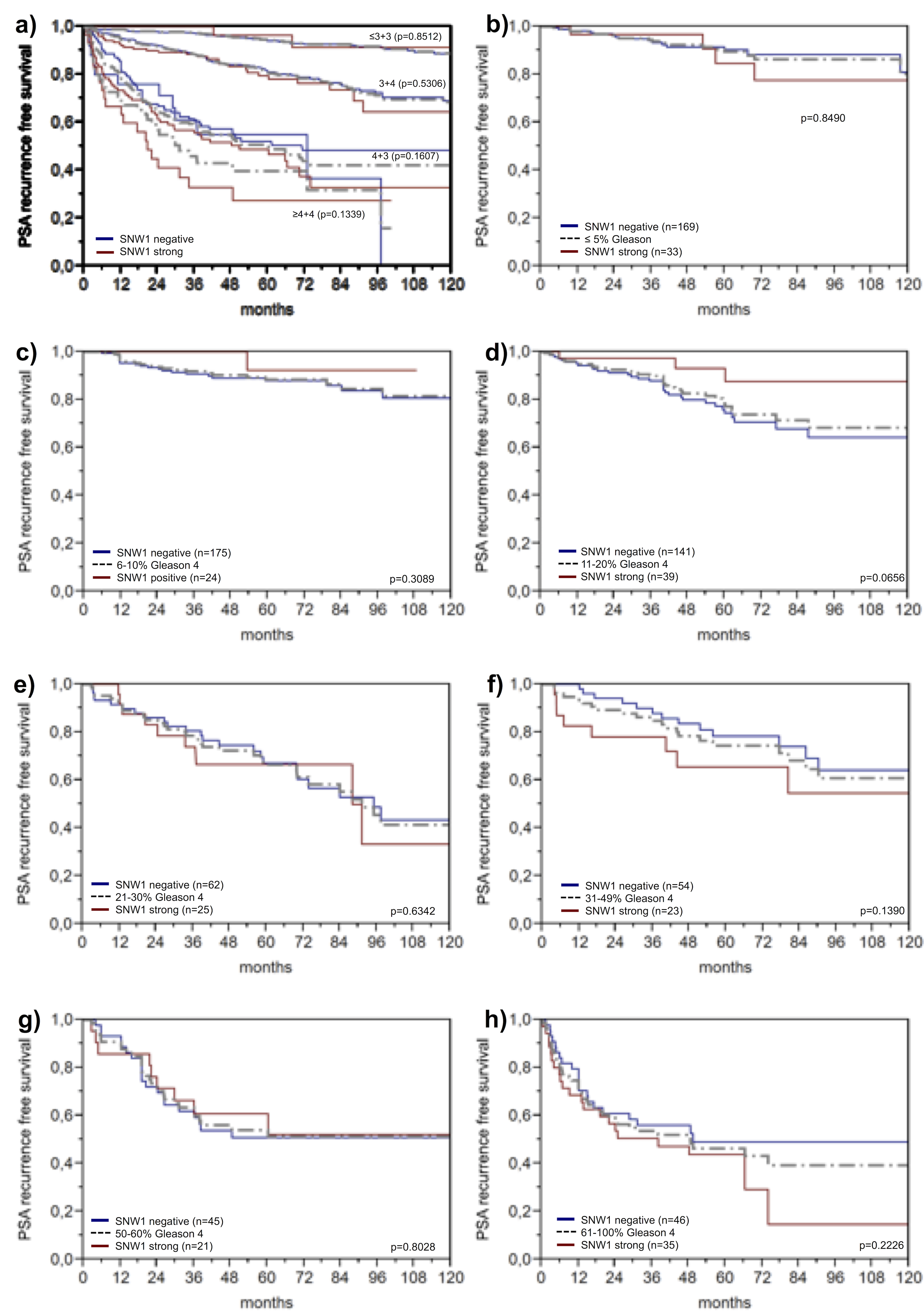


**Fig. S2** Prognostic impact of negative or strong SNW1 expression in subsets of cancers defined by a) the classical Gleason score (black dotted lines) and b-h) the quantitative Gleason score categories (black dotted lines) defined by the percentage of Gleason 4 patterns: b) ≤5%, c) 6-10%, d) 11-20%, e) 21-30%, f) 31-49 %, g) 50-60%, and h) 61-100% Gleason 4 pattern.
